# Flash glucose monitoring: objective, not self-referential, outcomes are needed. Reply to Warren RE [letter]

**DOI:** 10.1007/s00125-018-4652-9

**Published:** 2018-06-02

**Authors:** Jan Bolinder, Per Oskarsson, Ramiro Antuna, Petronella Geelhoed-Duijvestijn, Jens Krӧger, Raimund Weitgasser

**Affiliations:** 1 Department of Medicine, Karolinska University Hospital Huddinge, Karolinska Institute, 141 86 Stockholm, Sweden; 2 Department of Medicine, Clinica Diabetologica, Gijon, Spain; 3 Department of Internal Medicine, Haaglanden Medisch Centrum, Den Haag, the Netherlands; 4 Department of Diabetes, Zentrum für Diabetologie Hamburg Bergedorf, Hamburg, Germany; 5 Department of Medicine, Wehrle-Diakonissen Hospital, Salzburg, Austria; 6 1st Department of Medicine, University Hospital of Paracelsus, Medical Private University, Salzburg, Austria

**Keywords:** Clinical diabetes, Devices, Hypoglycaemia, Insulin therapy

*To the Editor:* We thank Dr Roderick Warren for his letter [1] regarding our subgroup analysis of the Novel Glucose-Sensing Technology and Hypoglycaemia in Type 1 Diabetes: a Multicentre, Non-masked, Randomised Controlled Trial (IMPACT; NCT02232698) [2]. In line with the accepted practice in continuous glucose monitoring studies, the trial was designed to compare individuals using the FreeStyle Libre system (Abbott Diabetes Care, Witney, UK) for glucose monitoring with those using the conventional technique, self-monitoring blood glucose (SMBG). The main glycaemic outcome measures with this type of study design require sensor glucose results to be visible to the intervention group but blinded in the control group. Warren reiterates a valid point regarding using the same device for the treatment and assessment of an outcome. This was indeed noted in our original study article [3]; however the general consensus is that there is no practical alternative to this approach [4].

With regard to the reliability of the sensor recordings, the accuracy of FreeStyle Libre in the low glucose range (capillary reference <4.4 mmol/l [80 mg/dl]), calculated from raw data collected by Bailey et al [5] had a mean bias of 0.2 mmol/l (2.9 mg/dl) and a mean absolute difference (MAD) of 0.6 mmol/l (10.4 mg/dl). In the IMPACT study baseline phase, mean bias was 0.0 mmol/l (0.3 mg/dl) and MAD 0.6 mmol/l (10.6 mg/dl) for capillary glucose <4.4 mmol/l. A recent head to head study supports the accuracy of FreeStyle Libre vs two continuous glucose monitoring (CGM) devices in the hypoglycaemic range [6].

Warren states that studies like this require externally valid outcomes such as HbA_1c_ or severe hypoglycaemia but does not suggest a viable alternative outcome that could have been utilised for this study. HbA_1c_ did not increase in the intervention group, which should be viewed positively in the context of a study population with extremely well-controlled blood glucose, reduced hypoglycaemia and a final mean HbA_1c_ of 53.0 mmol/mol. Severe hypoglycaemia is indeed very rare, with six instances reported in the treatment phase of this study (two in the intervention group and four in the control group), and therefore much larger numbers of patients would need to be studied over long periods to detect a clinically relevant improvement. Notably, there was a 30% reduction in the frequency of participant-reported symptomatic hypoglycaemia in the final 2 weeks (*p* = 0.0063). Hence, in our view, this similar reduction in symptomatic hypoglycaemia is corroborating evidence for the validity of the primary outcome result.

## Abbreviation

IMPACT: Novel Glucose-Sensing Technology and Hypoglycaemia in Type 1 Diabetes: a Multicentre Non-masked Randomised Controlled Trial
